# Necrotizing fasciitis following primary peritonitis caused by *Streptococcus pyogenes* with covS mutation in a healthy woman: a case report

**DOI:** 10.1186/s40981-019-0249-7

**Published:** 2019-04-27

**Authors:** Masashi Inoue, Eisuke Kako, Rie Kinugasa, Fumiaki Sano, Hironobu Iguchi, Kazuya Sobue

**Affiliations:** Department of Anesthesiology and Intensive Care Medicine, Nagoya City University Graduate School of Medical Sciences, 1-Kawasumi, Mizuho-cho, Mizuho-ku, Nagoya, 467-8601 Japan

**Keywords:** Primary peritonitis, Necrotizing fasciitis, *Streptococcus pyogenes*, CovS mutation

## Abstract

**Background:**

Primary peritonitis due to *Streptococcus pyogenes* (*S. pyogenes*) is uncommon in patients without comorbid conditions such as immunosuppression, nephritic disease, or liver cirrhosis. Furthermore, it does not cause another infection at the same time in a healthy person. However, several *S. pyogenes* mutants have been reported, and some of them exhibit strong virulence. Mutation of the control of virulence (cov) S gene of *Streptococcus* enhances bacterium survival by repressing negative regulators of virulence, which causes bacterial invasion of aseptic tissues, such as the parenteral space. We report a case of primary peritonitis and subsequent necrotizing fasciitis by the same *S. pyogenes* species with mutated covS in a previously healthy woman.

**Case presentation:**

We present the case of a 55-year-old woman admitted to the hospital due to abdominal pain and nausea. She was treated for peritonitis. A few days later, she became hypotensive and tachycardic and was transferred to the intensive care unit (ICU) for the treatment of septic shock with primary peritonitis. On the second day of her ICU stay, both of her forearms developed swelling and redness around the peripheral injection site. The patient had developed necrotizing fasciitis. Since her skin symptoms spread rapidly, urgent debridement was performed. Her condition improved with antibiotic treatment and multiple episodes of debridement. *S. pyogenes* was detected in cultures of the patient’s blood, ascites, and skin. The identified strain was emm89 genotype and had a genetic mutation of covS.

**Conclusions:**

*S. pyogenes* with covS mutation may spread from a portal, such as the upper respiratory tract or digestive system, to all organs immediately, causing septic shock. Infection with *S. pyogenes* with mutated genes should be considered in the differential diagnosis of gastrointestinal symptoms, even in a previously healthy patient.

## Background

Primary peritonitis is an acute infection of the peritoneum without an obvious causative source; it is uncommon in the absence of comorbid conditions, including immunosuppression, nephritic disease, or liver cirrhosis, particularly in healthy individuals [1]. Primary peritonitis can be caused by infection with *Enterobacteriaceae* (e.g., *Escherichia coli*, *Klebsiella* spp.), *Streptococcus pneumonia*, and *Enterococcus* spp.; however, it is rarely associated with *Streptococcus pyogenes*, which usually causes pharyngitis, erysipelas, and tonsillitis. Necrotizing fasciitis is a severe infection due to *S. pyogenes*, which has a high mortality. This infection generally occurs in the extremities around the gate of bacterial invasion in the skin such as trauma and surgical wound. It is very rare to develop necrotizing fasciitis secondary to primary peritonitis by the same bacteria species. Recent studies have suggested that *S. pyogenes* carrying mutations in covS gene can cause severe invasive infections. In this report, we describe the investigation and management of a 55-year-old woman with primary peritonitis and necrotizing fasciitis caused by *S. pyogenes* with a mutated covS.

## Case presentation

**Fig. 1:**
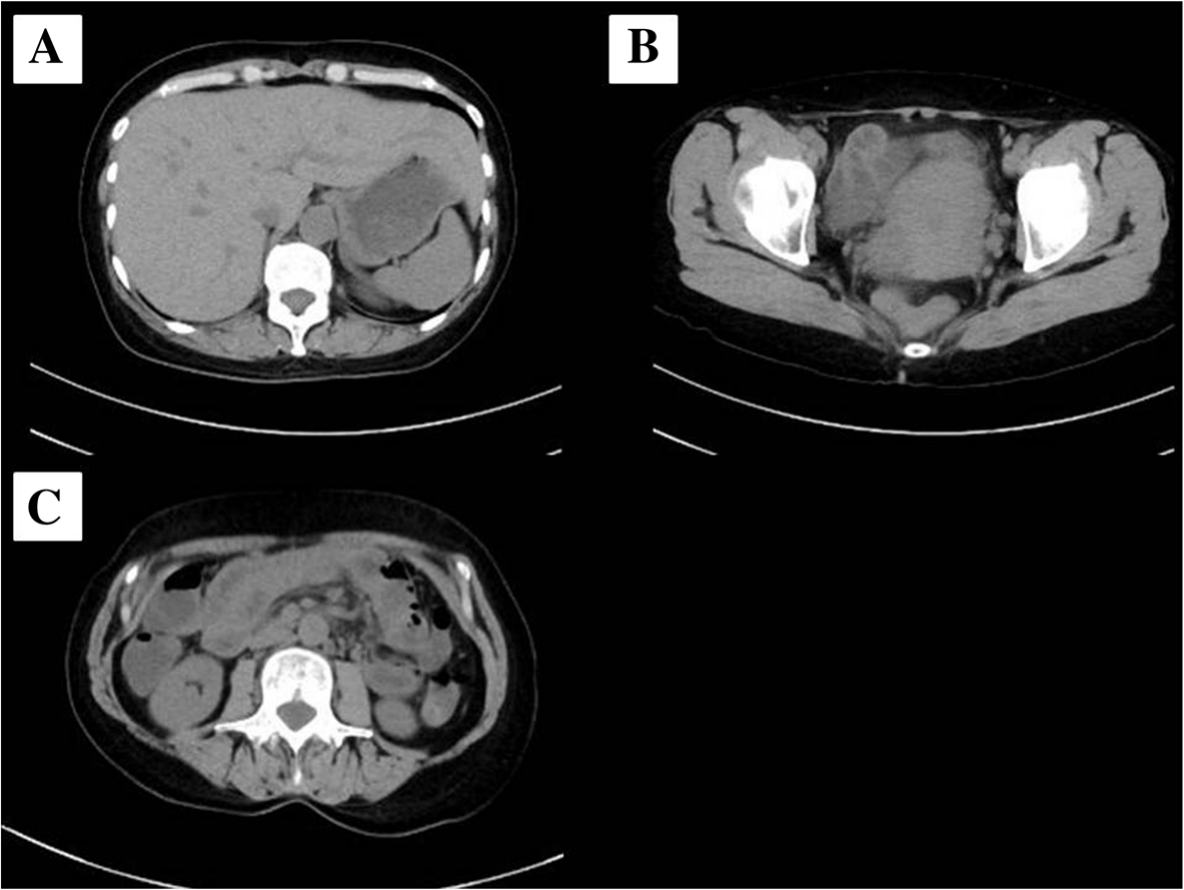
Abdominal computed tomography examination on admission. Abdominal computed tomography examination on admission showed no ascites (**a**, **b**) and a thickened wall throughout the entire small intestine (**c**)

**Fig. 2:**
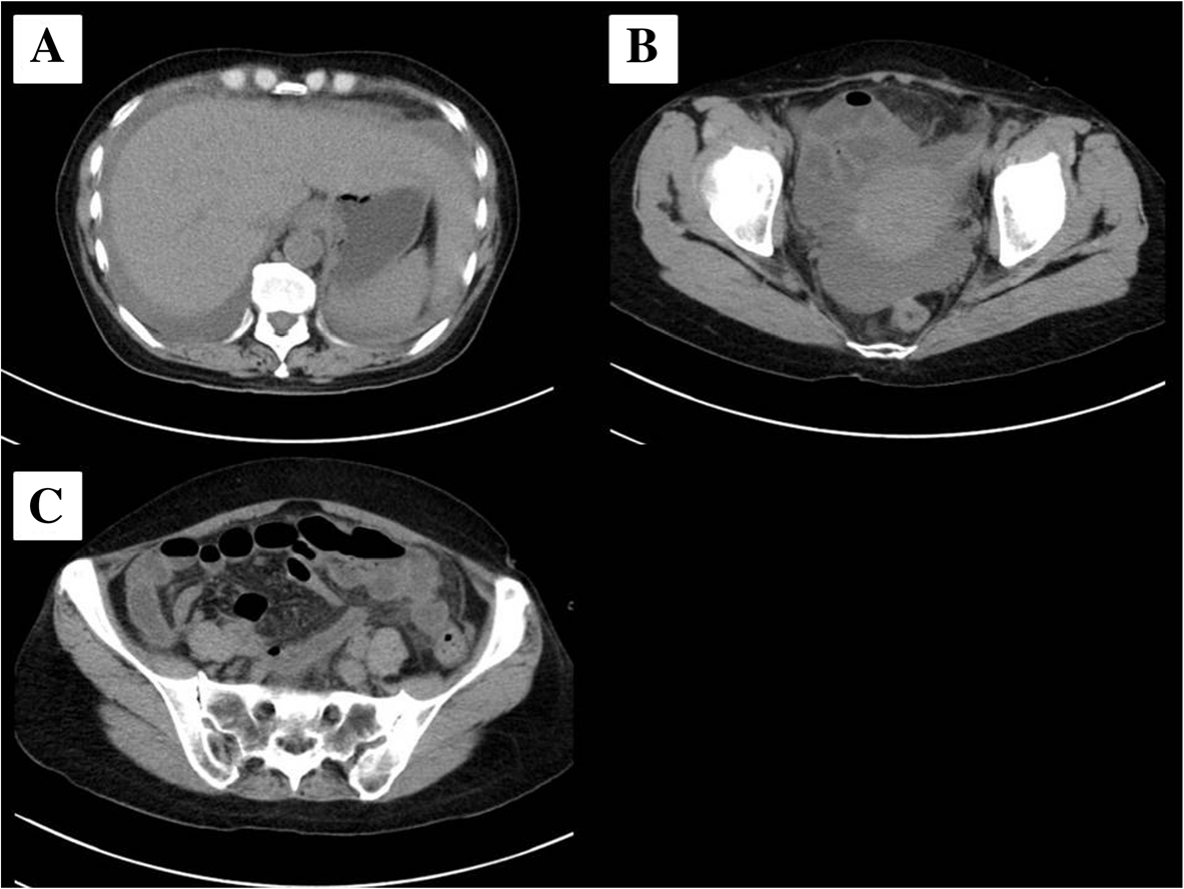
Computed tomography of the abdomen. Computed tomography of the abdomen detected ascites on the liver (**a**) and Douglas’s fossa (**b**). CT imaging also showed intestinal edema (**c**)

**Fig. 3:**
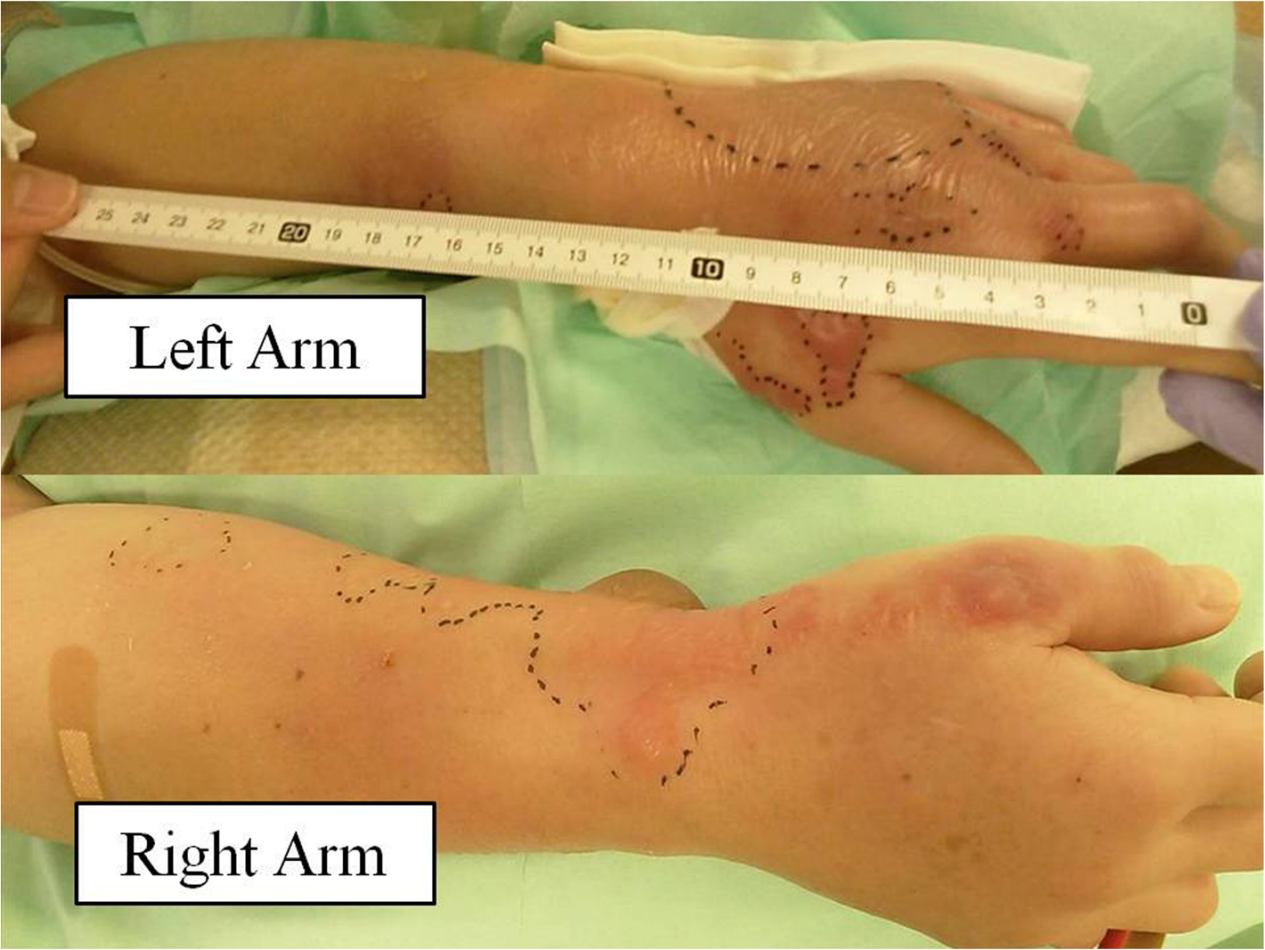
The patient’s both arms on ICU day 2. Both arms developed swelling and redness on ICU day 2. Dashed line shows the range of swelling

**Fig. 4:**
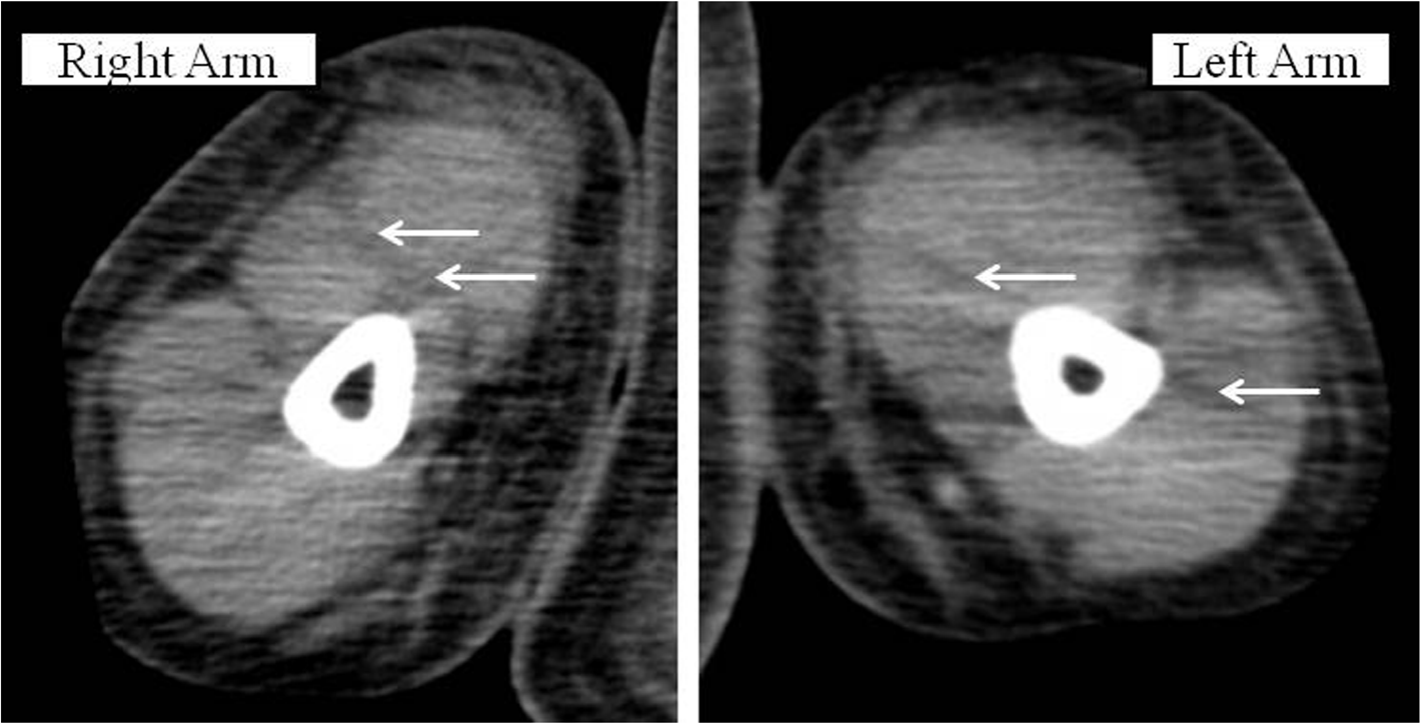
Computed tomography images of the forearms. Non-contrast CT images show edema between the muscles (white arrows)

A previously healthy 55-year-old woman entered a general ward complaining of lower abdominal pain and nausea. She was diagnosed with gastroenteritis and received fluid rehydration therapy with 1500 mL of hypotonic electrolyte solution per day, and intravenous acetaminophen was used for abdominal pain. Her nausea was improved after admission, so she did not take antiemetics. Her vital signs included a heart rate of 103/min, blood pressure of 104/67 mmHg, oxygen saturation of 97% while breathing ambient air, and temperature of 39.7 °C. Laboratory data showed a white blood cell count of 29.5 × 10^9^/L and C-reactive protein of 19.3 mg/dL. An abdominal computed tomography (CT) examination showed a thickened wall of the small intestine but no ascites (Fig. 1). She had no travel history and had not taken any medications or consumed any perishables. Any bacterial culture examination was not performed until ICU admission because infectious disease was not anticipated. Despite fluid rehydration therapy, her general condition worsened. On day 6, she entered the ICU because of disturbance of consciousness and respiratory failure. Before ICU admission, her vital signs were as follows: heart rate, 135/min; systolic blood pressure, 60 mmHg; and respiratory rate, 50 breaths/min. Laboratory data showed a white blood cell count of 10.6 × 10^9^/L and elevated C-reactive protein of 34.2 mg/dL. An abdominal CT showed intestinal edema and ascites on the liver and Douglas’s fossa (Fig. 2). Her limbs were cold, and both forearms were swollen because of the leakage of the peripheral infusion. Furthermore, she had respiratory distress. Septic shock was suspected, and she was treated with fluid resuscitation, empirical antibiotics (meropenem, linezolid, and micafungin), and a vasopressor. On ICU day 2, her arms became swollen and red (Fig. 3). CT of the upper limbs showed edema between the muscles, suggesting necrotizing fasciitis (Fig. 4). Therefore, emergency skin incision and debridement were performed. A pathological examination showed intense polymorphonuclear cell infiltration with diffuse connective tissue necrosis, compatible with a diagnosis of necrotizing fasciitis. Renal replacement therapy was also initiated because of acute kidney injury. Serum creatinine had increased from 0.72 to 3.03 mg/dL (stage 3 based on the KDIGO classification). Surprisingly, *S. pyogenes* was detected from cultures of ascitic fluid, which were taken before sepsis developed, from blood and skin. The empirical antibiotics were switched to penicillin G, linezolid, and clindamycin (linezolid was discontinued on ICU day 8). On ICU day 3, 1420 mL of ascites was drained and the patient underwent second debridement of the upper limbs, because the inflammation had extended over the shoulders. Her condition improved, and she overcame the septic shock on ICU day 6. On ICU day 9, *Pseudomonas aeruginosa* and *Candida parapsilosis* were found in a skin culture. Therefore, the antibiotics were switched to ciprofloxacin and micafungin. Flap angioplasties of both forearms were performed on ICU day 22. The pedicle flaps were harvested from each intact area of the arms and were placed to defect sites of the respective limbs, then additional skin grafting was performed on ICU day 36. The patient was transferred from the ICU to the general ward on day 62 (ICU day 57). She was discharged to a regional medical support hospital on day 110.

A genetic examination of the *S. pyogenes* harvested from the skin and blood cultures showed that the strain had a mutation in the covS gene.

## Conclusions

There are some reports of peritonitis caused by *S. pyogenes* infection in healthy women [2–7]. The predominance of women among *S. pyogenes* peritonitis patients might implicate ascending infections of the genitourinary tract, since *S. pyogenes* are often part of the vaginal flora [8]. According to a systematic review of primary peritonitis caused by streptococcus, 16% of patients developed streptococcal toxic shock syndrome (STSS) from an ascending infection originating from the vagina, 9% of patients developed it from a droplet infection, 6% of patients developed it from pharyngitis, and 69% of patients developed it via an unknown route [9]. In our case, we could not determine the primary site of infection; we should have collected a vaginal culture to confirm whether the infection originated in the vaginal tract. For the same reason, we should have collected a pharynx culture. The diagnosis of peritonitis was based on the presence of purulent ascites cultures with increased lactate dehydrogenase (LDH) and white blood cells (WBC) and CT findings of increased intensity within the peritoneum and smooth, uniform thickening of the small intestine wall.

Necrotizing fasciitis is an infection of the deeper subcutaneous tissue and fascia that is characterized by rapidly spreading necrosis of the skin. The successful management of necrotizing fasciitis is dependent on early recognition, but classical signs of a necrotizing process are not initially apparent. If the patients have a defined portal of entry, such as a surgical incision, burn, insect bite, or varicella lesion, the skin around will be red with severe pain and swelling. However, in the 50% of patients who develop necrotizing fasciitis without a defined portal of entry, the infection begins deep in the tissues, frequently at the site of a hematoma, muscle strain, or traumatic joint injury [1]. There are some studies of necrotizing fasciitis associated with drug injection [10, 11]. One study found that 54% of drug users with necrotizing fasciitis developed the infection at an injection site [10]. The same study showed that *S. pyogenes* was the third most frequently detected gram-positive aerobe, following *Staphylococcus aureus* and viridans streptococci. Another study revealed that patients with a necrotizing fasciitis after injection or infiltration therapy have a poor prognosis in terms of survival and amputation rate [11]. In our case, redness and swelling spread from peripheral infusion cannulation sites on both forearms. Thus, the *S. pyogenes* infection might have originated from the injection sites. However, it is considered that the necrotizing fasciitis was spread from primary peritonitis via bloodstream infection which caused septic shock at ICU admission. The reason is that *S. pyogenes* species which had the same sensitivity to antibiotics were detected from ascites, blood, and skins in both arms. Then, as of ICU admission, there were no inflammation signs in the limbs even though peritonitis and sepsis had already occurred. Furthermore, she developed necrotizing fasciitis concurrently in both arms which were apart from each other. It seems questionable if the bacteria involved in the fasciitis invaded from the skin.

Additionally, this *S. pyogenes* lineage comprised emm89-type strains and carried a mutation in covS gene. This group produces many surface-bound and extracellular virulence factors that contribute to pathogenesis in complex ways. CovS/CovR is a key two-component regulatory system of gene transcription in *S. pyogenes* [12–14]. CovS/CovR regulates the expression of 15% of the genes of *S. pyogenes* [15, 16], including streptococcal pyrogenic exotoxin B (SpeB). Because the expression of active SpeB causes the degradation of secreted GAS (group A streptococci) proteins, including several virulence factors, reduced SpeB expression in covS mutants confers strong virulence on *S. pyogenes* [17, 18]. Recent studies have also described an inverse relationship between disease severity and the degree of a cysteine protease, SpeB [12, 13]. Moreover, a previous report has suggested that mutated covS plays important roles in the in vivo dissemination of *S. pyogenes* in humans from the upper respiratory tract to aseptic tissues, such as blood and cerebrospinal fluid [19], and some studies have described the significance of covS mutations in the pathogenesis of STSS [20, 21]. In this case, it was considered that this covS mutation contributed to the development of peritonitis in a previously healthy patient.

In our case, the *S. pyogenes* was sensitive to penicillin G. Therefore, we used penicillin G and clindamycin as definitive therapies. We also used linezolid until the pathogen was identified because the possibility of community-acquired methicillin-resistant *Staphylococcus aureus* (MRSA) involvement could not be ruled out. The guidelines of the Infectious Disease Society of America recommend the use of penicillin plus clindamycin for the treatment of documented streptococcal necrotizing fasciitis [22]. This recommendation is based on the finding that all strains of *S. pyogenes* are sensitive to penicillin and that clindamycin suppresses *S. pyogenes* exotoxin and M protein production. M protein is an important molecule for adhering to host cells; furthermore, it has anti-opsonin activity, which is associated with resistance to immunity. Moreover, the guidelines show that empirical treatment of polymicrobial necrotizing fasciitis should include agents that are effective against both aerobes, including MRSA, and anaerobes.

In summary, we report a rare case of secondary necrotizing fasciitis following primary peritonitis with a *S. pyogenes* infection in a previously healthy woman. *S. pyogenes* primary peritonitis should be considered in the differential diagnosis of gastrointestinal symptoms, even in healthy women. In these cases, it is important to detect the pathogen underlying peritonitis through abdominocentesis or a diagnostic laparotomy. Subsequently, antibiotic therapy should be initiated promptly. Because *S. pyogenes* with mutated covS may spread immediately from the upper respiratory tract, the vagina, or a peripheral injection site to the organs, rapid diagnosis and treatment are required. Furthermore, it is important to avoid unnecessary catheter placement to prevent catheter-related blood stream infection.

## Abbreviations

Cov: Control of virulence
CT: Computed tomography
GAS: Group A streptococci
ICU: Intensive care unit
MRSA: Methicillin-resistant *Staphylococcus aureus*
*S. pyogenes*: *Streptococcus pyogenes*
SpeB: Streptococcal pyrogenic exotoxin B
STSS: Streptococcal toxic shock syndrome
